# Native Tricuspid Valve Infective Endocarditis After Breast Skin Abscess

**DOI:** 10.7759/cureus.46607

**Published:** 2023-10-06

**Authors:** Sumayya Sami, Faisal Ali, Kamran Pasha

**Affiliations:** 1 Department of Internal Medicine, Aga Khan University Hospital, Karachi, PAK; 2 Department of Gastroenterology and Hepatology, Shifa International Hospital Islamabad, Multan, PAK; 3 Department of Acute Medicine, Royal Surrey County Hospital, Guildford Surrey, GBR

**Keywords:** transthoracic echocardiography (tte), skin abscess, methicillin resistant staphylococcus aureus (mrsa), non-intravenous drug users, tricuspid valve infective endocarditis

## Abstract

Tricuspid valve infective endocarditis is a rare disease in non-intravenous drug users. It can occur with congenital heart disease, foreign bodies such as central venous catheters and intracardiac devices, and in immunocompromised patients. In the present case, there was a left-sided breast abscess associated with tricuspid valve endocarditis in a patient without any apparent underlying causative factors.

We present a case of a young female in her early 20s who arrived at the emergency department with complaints of fever, epistaxis, and vomiting. On clinical examination, she was found to have a fading 2 cm pinkish left breast skin lesion, which had formed on her breast 10 days ago. Blood cultures identified methicillin-resistant *Staphylococcus aureus *in the blood. A CT scan of the chest, abdomen, and pelvis revealed splenomegaly and an infective focus in the spleen. Subsequent echocardiography confirmed the diagnosis of infective endocarditis of the native tricuspid valve, which was treated with intravenous vancomycin. There was no history of intravenous drug abuse, congenital heart disease, placement of an intracardiac device, central venous catheter, or an immunocompromised state in this patient. Therefore, the diagnosis of infective endocarditis, characterized by a native tricuspid valve vegetation identified as a consequence of a left breast skin abscess, was made.

A high index of suspicion is required for a non-specific presentation of tricuspid valve infective endocarditis and in the absence of any prior history of risk factors for right-sided infective endocarditis. Timely initiation of antibiotics depends on a preliminary clinical diagnosis.

## Introduction

Infective endocarditis is a disease that involves the endocardial surface of the heart, more commonly affecting the heart valves [1]. While right-sided infective endocarditis is much less common than left-sided infective endocarditis, accounting for 5% to 10% of all infective endocarditis cases and usually occurs with a predisposing risk factor [2], patients presenting with no primary cause have also been described [3-5]. The present case involved a left-sided breast abscess associated with tricuspid valve endocarditis. Diagnosing patients without a history of intravenous drug abuse and no underlying causative factors can be difficult and poses a significant clinical challenge.

## Case presentation

A young woman in her early 20s presented with a five-day history of fever, epistaxis, and vomiting. She denied any history of diarrhea, headache, joint pains, or chest symptoms but did report episodes of hematuria. She had no exposure to cattle, sheep, goats, or hares and had not recently traveled abroad. She did not have a significant medical history and was not taking any regular medications. She lived with her partner, had no children, and was a housewife. She had no history of smoking, alcohol use, or intravenous drug abuse.

On examination, she was noted to be febrile with a temperature of 38.6°C. The initial examination was unremarkable apart from blood staining on her left nostril and a regular heart rate of 107 bpm, respiratory rate of 19 breaths/min, oxygen saturation of 98% on room air, and a blood pressure of 126/80 mm Hg. There was a 2-cm splenomegaly, the chest was clear, no murmur was noted, and there were no rashes on her body.

ECG showed sinus tachycardia of 110 bpm. Initial blood tests revealed a hemoglobin level of 118 g/L (115-165 g/L), a white cell count of 9.3 × 109/L (4.0-11.0 × 109/L), and a platelet count of 302 × 109/L (150-400 × 109/L), with negative results for dengue IgM antibody, Crimean-Congo virus RNA PCR, and blood film for malaria. The chest X-ray was clear. Urinalysis was negative for protein, leucocytes, and nitrites but showed 4+ blood, and her serum beta-human chorionic gonadotropin level was <2.0 mIU/mL (<10 mIU/mL). Three sets of blood cultures were sent. Her serum CRP was 82.90 mg/L (0-14 mg/L), with a procalcitonin of 0.63 ng/mL (<0.5 ng/mL). She had an ultrasound of the upper abdomen suggestive of an enlarged spleen measuring 160 mm with a cystic lesion within it.

The patient’s symptoms and a normal full blood count were initially thought to result from a platelet qualitative defect. Von Willebrand disease (VWD) was considered as a possible cause; however, although a normal platelet count is usually seen in VWD, a normal plasma von Willebrand factor of 220% (50-160%) virtually excluded it [6]. Furthermore, the patient had a normal bleeding time of 1.15 min (1-7 min) and a normal activated partial thromboplastin time of 23.9 s (22.9-34.5 s).

**Figure 1:**
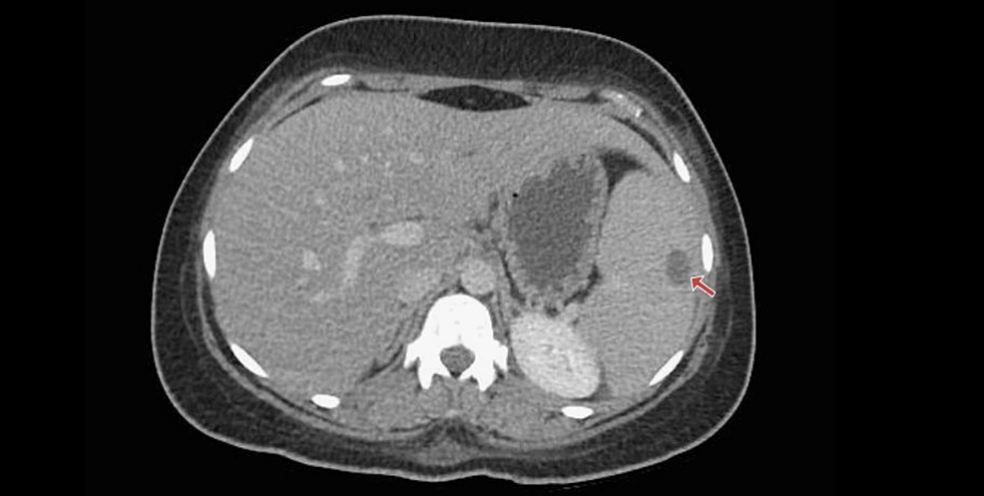
CT of the abdomen shows a hypodense focus in the spleen

**Figure 2:**
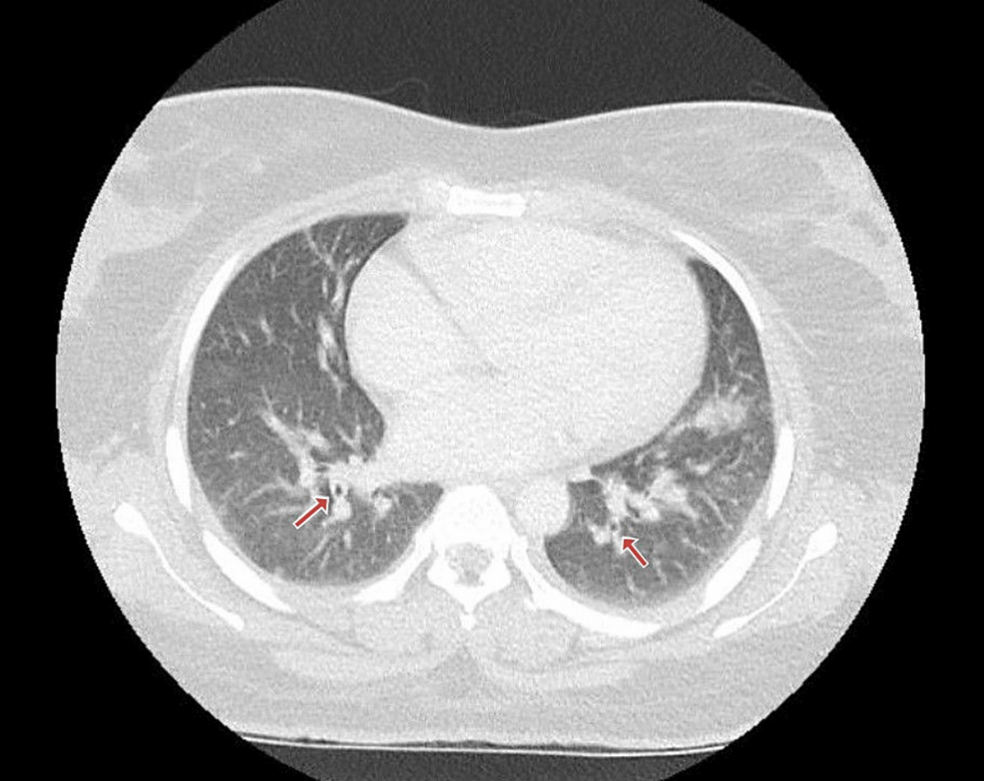
CT of the chest shows multiple cavitary nodules in bilateral lungs

After admission to the ward, a CT scan of the chest, abdomen, and pelvis (CT CAP) was performed, which confirmed splenomegaly with an infective focus suggestive of splenic abscess (Figure 1), along with multiple cavitary nodules in bilateral lungs (Figure 2).

**Figure 3:**
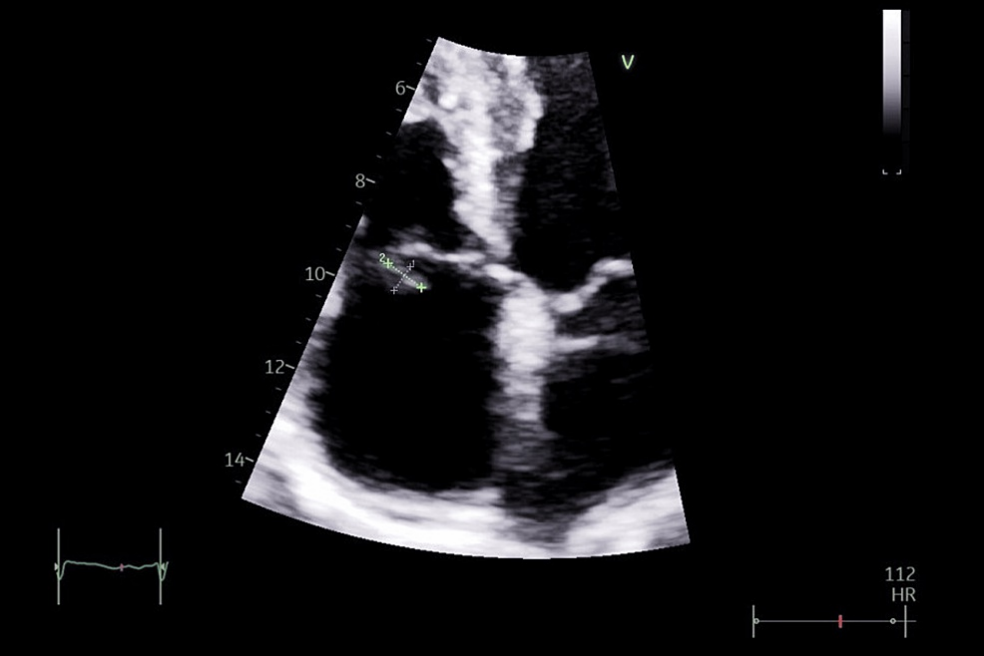
TTE image showing a vegetation on the anterior leaflet of the tricuspid valve

Her autoimmune screen, including antinuclear antibody (ANA) and antineutrophil cytoplasmic antibodies (ANCA), was negative. Serum fungal markers, including beta-D-glucan 10 pg/mL (<60 pg/mL) and galactomannan 0.17 (<0.5), were negative. She was also tested for HIV and was found to be negative. In the meanwhile, her two sets of blood cultures showed the growth of methicillin-resistant *Staphylococcus aureus* (MRSA). On further testing, the transthoracic echocardiography (TTE) revealed an oscillating mass, measuring 12 × 6 mm, compatible with a vegetation on the anterior leaflet of the tricuspid valve (Figure 3). There was mild tricuspid regurgitation with the normal size of right atrium and right ventricle.

Upon re-examination of the patient, a fading, non-discharging, soft, painless skin lesion was noted on her left breast. The lesion had a smooth surface, was slightly raised above the surrounding skin, measured 2 cm in size, was pinkish in color, and had initially been filled with pus when it formed, developing on her breast 10 days ago. On further questioning, the patient provided no history of congenital heart disease, placement of intracardiac devices, central venous catheters, or an immunocompromised state. As mentioned earlier, there was no history of intravenous drug abuse. A diagnosis of infective endocarditis with a native tricuspid valve secondary to a left breast skin abscess was made. She developed septic pulmonary emboli [7], which have been observed in patients with native tricuspid valve endocarditis without underlying risk factors [8].

After the initial course of supportive treatment in the emergency department, which included intravenous antipyretics, antiemetics, and hydration with 1 L of Ringer's lactate, the patient was treated with empirical antibiotics. Blood cultures were sent, and the patient received 1000 mg of intravenous vancomycin and 2000 mg of intravenous ceftriaxone, following the local treatment protocol for sepsis. This treatment was continued with 1000 mg of intravenous vancomycin every 12 hours and 2000 mg of intravenous ceftriaxone once daily until the final reports of blood cultures were disclosed.

She remained consistently febrile during the first 24 hours of admission, despite receiving empirical antibiotics, antipyretics, and hydration with lactated Ringer's solution at a rate of 100 mL/h. The patient reported intermittent vomiting; therefore, the hydration rate was increased to 125 mL/h, and a regular antiemetic was added every eight hours.

Throughout this treatment, the fever started to decrease in frequency but did not completely resolve until day 3 of her admission. As soon as her blood cultures were processed, and gram-positive cocci were reported, intravenous ceftriaxone was stopped, and intravenous vancomycin was continued. Intravenous gentamicin at 80 mg every eight hours was started, which was later discontinued once the sensitivities in blood cultures were finalized.

The patient's symptoms rapidly resolved after the initiation of antibiotics. Moreover, vancomycin levels were checked after the fourth dose (trough levels), 30 minutes prior to the scheduled dose, and were found to be 4.40 mg/L (10-20 mg/L). Therefore, the vancomycin dosage was increased from every 12 hours to every eight hours for a duration of six weeks. An uneventful placement of a peripherally inserted central catheter (PICC) was performed in her right arm, guided by ultrasound and under local anesthesia, starting from the right basilic vein and extending into the lower third of the superior vena cava. Blood cultures were repeated, and she was discharged with final reports to be reviewed in the clinic during an early follow-up. Before discharge, the patient was asymptomatic and reported that her hematuria and epistaxis had resolved.

The patient continued to receive intravenous vancomycin and intravenous fluids for five days, which led to the resolution of sepsis. The infective endocarditis in this case was deemed to be caused by her left breast skin abscess, which was in the resolving phase when she arrived at the emergency department with sepsis symptoms. Upon examination, there were no track marks on her arms that might suggest intravenous drug abuse. Auscultation of the precordium and echocardiography ruled out any underlying congenital heart disease. Further investigations confirmed the absence of an immunocompromised state, such as HIV, as her HIV test was non-reactive; diabetes mellitus, as her plasma fasting glucose was 5.2 mmol/L (<6.0 mmol/L), and postprandial glucose after two hours of a meal was 5.6 mmol/L (<7.8 mmol/L); autoimmune conditions, as her ANA and ANCA profiles were negative; malignancy, as her full blood count was normal and CT CAP did not demonstrate any malignant lesion; and other congenital or acquired chronic illnesses, as her baseline renal function tests, liver function tests, and serum electrolytes were normal, and the examination was unremarkable except for the left-sided breast skin lesion. A skin biopsy was not performed as the lesion was regressing.

**Figure 4:**
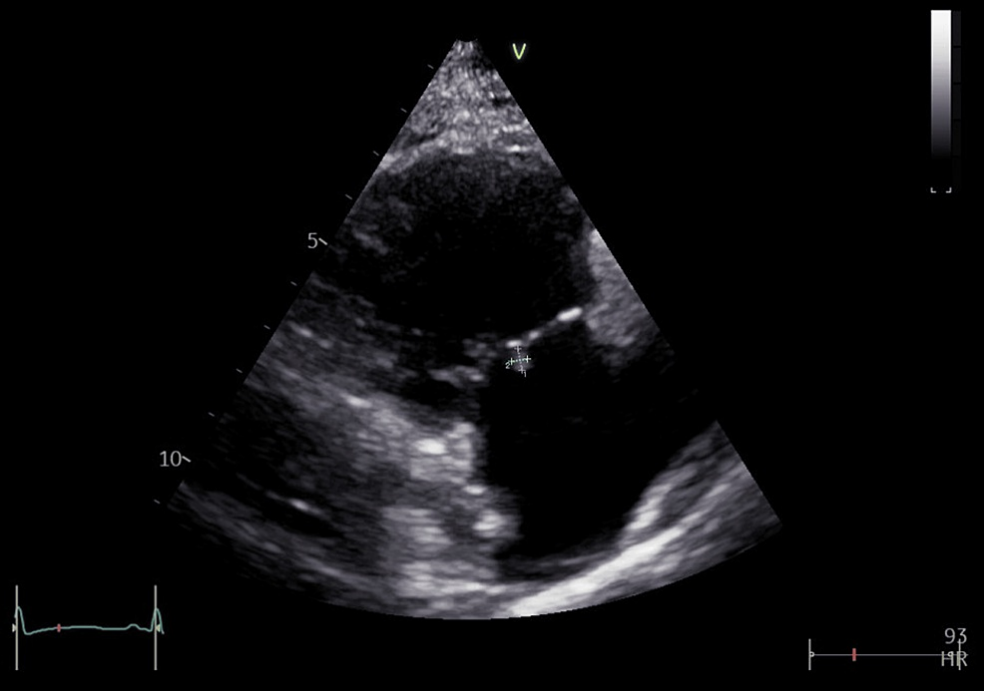
TTE image showing a small vegetation on the tricuspid valve

The patient was discharged with intravenous vancomycin at a dose of 1000 mg three times a day [9] via a PICC line, with monitoring of serum vancomycin levels to keep them in the therapeutic range. The use of initial low-dose gentamicin in addition to vancomycin for MRSA bacteremia and native valve endocarditis has been discouraged due to the risk of nephrotoxicity [10]. Repeated blood cultures taken at discharge revealed no bacterial growth, her serum CRP subsequently improved, and her skin lesion continued to get better and eventually resolved as she was followed up in the infectious disease clinic. Furthermore, TTE was repeated after six weeks of antibiotic therapy, which showed persistence of the tricuspid valve vegetation, measuring 4 × 3 mm, although its size had decreased (Figure 4). Complete resolution of vegetation is uncommon by the end of antimicrobial therapy, even with successful treatment [11].

She did not receive any blood transfusion during her hospital stay or later on during follow-up, as her hemoglobin remained stable throughout, despite experiencing recurrent episodes of hematuria and epistaxis as symptoms of infective endocarditis.

There are certain limitations in the case detailed above. The patient did not undergo sputum culture and bronchoscopy for bilateral pulmonary cavitary lesions during the hospital admission, as microorganisms other than MRSA were not suspected, given that her symptoms improved with the initiation of treatment for infective endocarditis. Second, the splenic abscess, as reported on CT CAP, measured nearly 21 × 14 mm, and no particular intervention was performed for it besides continuing antibiotics, as once again, the fever subsided with intravenous antibiotics. A repeat radiological investigation was later scheduled as an outpatient; however, the patient did not attend the further radiological investigations.

## Discussion

To establish a diagnosis of infective endocarditis definitively, clinicians have widely used the modified Duke criteria, which indicate that a patient must meet one of the following: two major criteria, one major criterion with three minor criteria, or five minor criteria [12]. Our patient fulfilled two major and three minor criteria. Nonetheless, in most cases of tricuspid valve infective endocarditis, empiric antibiotics are initially used for treatment, and research has shown that in 70-85% of cases, antibiotics alone can eradicate the bacteremia. Furthermore, adjusting the antibiotic regimen after obtaining the culture sensitivity results has been shown to yield better outcomes [1].

Native tricuspid valve infective endocarditis is most frequently caused by intravenous drug abuse, intracardiac devices, or the placement of central venous catheters [2]. However, when observed in the absence of the abovementioned risk factors, accompanied by a bleeding history as manifested in this case, the clinician can be distracted from infective endocarditis. This distraction risks leading to a delay in diagnosis and possible serious morbidity and mortality.

The hematuria was explained by glomerulonephritis, a renal pathology commonly seen in patients with infective endocarditis [13]. However, epistaxis is a very rare complication or presenting symptom of left-sided infective endocarditis [14] and is extremely rare with right-sided infective endocarditis. Other potential causes of intermittent hematuria and epistaxis with fever, such as viral hemorrhagic fever including Crimean-Congo hemorrhagic fever and dengue, were considered. In this case, the patient had negative results for Crimean-Congo virus RNA PCR and dengue IgM antibody, respectively. Wegener's granulomatosis (WG) can present with similar symptoms along with pulmonary cavitary nodules; however, her ANCA test was negative. ANCA is typically present in nearly every patient with active and severe WG disease [15]. Furthermore, there was no proteinuria in her urinalysis, and the renal function tests were unremarkable; therefore, a definitive diagnostic procedure like renal biopsy was not performed [16].

*Staphylococcus aureus* is the most common organism causing infective endocarditis of the tricuspid valve in patients with underlying risk factors [2]. It also remains the most common pathogen in rare reports where tricuspid valve infective endocarditis occurred following psoas abscess [8], septic arthritis [4], septic abortion [17], or in cases with no clear predisposing condition [3,5]. Similarly, in our case, *Staphylococcus aureus* was the culprit pathogen following a left-sided breast skin abscess.

In addition, it should be noted that surgical intervention is required in patients with any of the following conditions: tricuspid vegetation greater than 20 mm with septic pulmonary emboli, bacteremia persisting for one week despite adequate treatment with antibiotics, or right-sided heart failure due to severe tricuspid regurgitation [18]. In the present case, there were no surgical indications, and timely treatment with intravenous vancomycin was the most appropriate step required to prevent the progression of the disease.

Finally, anticoagulation therapy is used for the treatment of non-infective pulmonary embolism, but it should not be instituted in cases of septic embolization due to the higher chances of bleeding in the region of the infected embolus. Therefore, eradication of infection is the key to the management of septic embolization [19]; consequently, no anticoagulation was used in the described case.

## Conclusions

This case describes a non-specific presentation of tricuspid valve infective endocarditis. A high index of suspicion is needed in patients with no history of risk factors for right-sided infective endocarditis. Prompt initiation of antibiotics is essential based on the preliminary clinical diagnosis.
